# Liver abscess in advanced hepatocellular carcinoma after atezolizumab plus bevacizumab treatment: A case report

**DOI:** 10.1097/MD.0000000000030486

**Published:** 2022-09-02

**Authors:** Keisuke Uchida, Yoshinori Ozono, Naomi Uchiyama, Hiroshi Hatada, Kenichi Nakamura, Yuri Komaki, Hisayoshi Iwakiri, Satoru Hasuike, Kenji Nagata, Yuichiro Sato, Hiroshi Kawakami

**Affiliations:** a Divison of Gastroenterology and Hepatology, Department of Internal Medicine, Faculty of Medicine, University of Miyazaki, Japan; b Department of Diagnostic Pathology, Division of Pathology, University of Miyazaki Hospital, Japan.

**Keywords:** atezolizumab, bevacizumab, liver abscess, *Raoultella ornithinolytica*

## Abstract

Hepatocellular carcinoma (HCC) is the sixth most common type of cancer globally. Since 2020, combination treatment with atezolizumab and bevacizumab were approved in patients with unresectable HCC in Japan, and atezolizumab plus bevacizumab is the first-line treatment for unresectable HCC.

**Patient concerns:**

A 73-year-old Japanese man diagnosed with a large HCC was treated with atezolizumab plus bevacizumab. After 2 cycles, he had fever and fatigue and was admitted to the hospital.

**Diagnosis:**

Abdominal contrast-enhanced computed tomography revealed tumor necrosis in HCC with gas formation in the necrotic area. Laboratory examination revealed a white blood cell (WBC) count of 16,340/μL and C-reactive protein (CRP) level of 33.0 mg/dL. Based on the above findings, he was diagnosed with a liver abscess.

**Interventions:**

Percutaneous transhepatic liver abscess drainage and broad-spectrum antibiotics treatment were performed.

**Outcomes:**

Despite liver abscess drainage, persistent fever and no improvement in the WBC count or CRP level was observed. The patient’s respiratory condition and renal function gradually worsened; The patient’s general condition did not improve despite the ventilator support and continuous hemodiafiltration, and he died on day 37.

**Lessons:**

We report the first case of liver abscess after atezolizumab plus bevacizumab treatment for unresectable HCC.

## 1. Introduction

Hepatocellular carcinoma (HCC) is the sixth most common type of cancer worldwide.^[1]^ Although early-stage HCC (Barcelona Clinic Liver Cancer [BCLC] stage 0 or A) may be curable by surgical resection, local ablation, and liver transplantation, intermediate (BCLC stage B) and advanced (BCLC stage C) stage HCCs are unresectable and treated by transarterial chemoembolization (TACE) and/or systemic treatment.

The multikinase inhibitors sorafenib and lenvatinib are the first-line systemic chemotherapy for unresectable HCC. Sorafenib showed a significant survival benefit compared to placebo in phase 3 trials,^[2,3]^ whereas lenvatinib showed non-inferiority to sorafenib in a phase 3 trial.^[4]^ Combination treatment with atezolizumab, an immune checkpoint inhibitor, and bevacizumab, an angiogenesis inhibitor, showed survival benefit in patients with unresectable HCC. The randomized controlled trial of sorafenib^[5]^ was approved in September 2020 and is currently the first-line treatment for unresectable HCC in Japan.

Herein, we report a case of extensive necrosis of HCC with a liver abscess after atezolizumab plus bevacizumab treatment for large unresectable HCC. To our best knowledge, this is the first report of the development of a liver abscess in a necrotic area after atezolizumab plus bevacizumab treatment for unresectable HCC.

## 2. Case presentation

**Figure 1.**
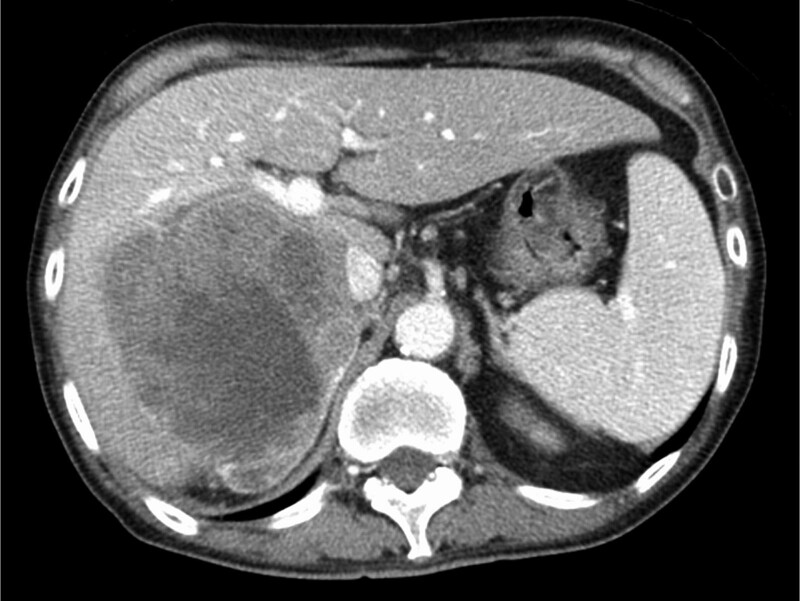
Abdominal computed tomography showing large hepatocellular carcinoma before atezolizumab plus bevacizumab treatment.

**Figure 2.**
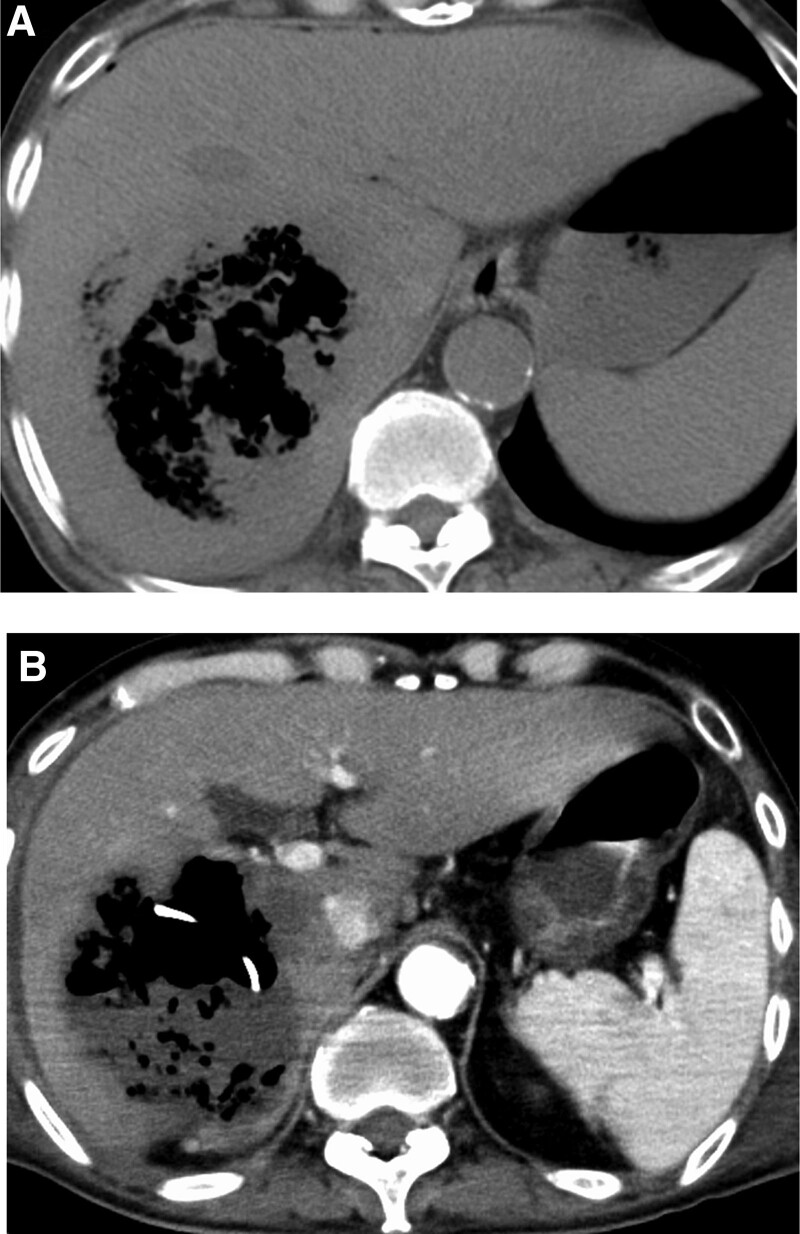
Abdominal computed tomography showing a gas-forming pyogenic liver abscess after atezolizumab plus bevacizumab treatment and (A) before and (B) after percutaneous liver abscess drainage.

A 73-year-old Japanese man diagnosed with a large right-lobe (11-cm in diameter) unresectable HCC (Fig. 1) was treated with atezolizumab plus bevacizumab. After 2 cycles, he had fever and fatigue and was admitted to the hospital. Abdominal contrast-enhanced computed tomography (CT) revealed extensive tumor necrosis in HCC, with gas formation in the necrotic area (Fig. 2). Additionally, common bile duct (CBD) stones were presented, but no dilation of the intrahepatic bile duct (IHBD) to CBD was observed. Laboratory examination revealed a white blood cell (WBC) count of 16,340/μL and C-reactive protein (CRP) level of 33.0 mg/dL. He was diagnosed with a liver abscess and referred to our hospital. His vital signs were as follows: blood pressure, 120/56 mmHg; pulse rate, 84 beats/min; temperature, 38.5°C; and Glasgow coma scale, E4V4M6. Laboratory data on admission showed a WBC count of 9600/μL, CRP level of 29.0 mg/dL, platelet count of 50 × 10^3^/μL, fibrin degradation product of 30.6 μg/mL, and prothrombin time of 53.4%. Based on these findings, disseminated intravascular coagulation (DIC) was performed.

Meropenem and metronidazole for the liver abscess and recombinant thrombomodulin for DIC were administered. Subsequently, percutaneous liver abscess drainage was performed, and approximately 100 mL of foul-smelling pus was observed; however, most of the components were gases. Although pus cultures indicated the presence of *Raoultella ornithinolytica*, blood culture tests were negative. Based on pus culture and drug sensitivity results, metronidazole was changed to vancomycin. Biliary infection associated with CBD stones was suspected to have caused the liver abscess; however, endoscopic retrograde cholangiopancreatography could not be performed because of the presence of DIC.

**Figure 3.**
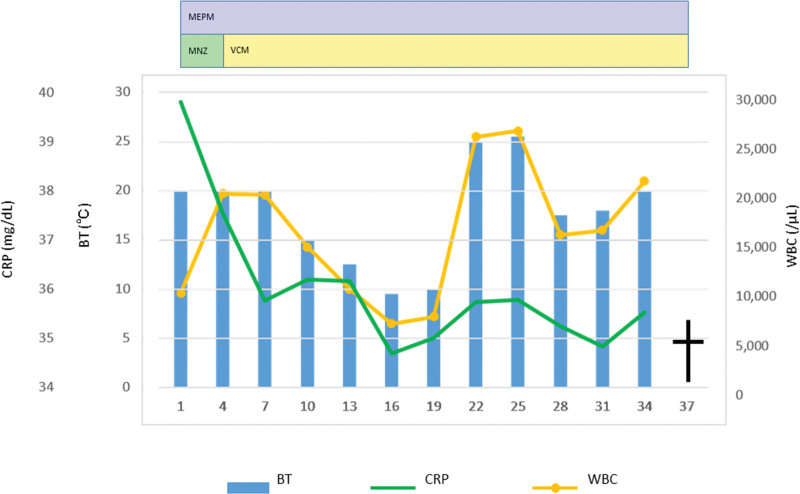
Clinical course. CRP = C-reactive protein, MEPM = meropenem, MNZ = metronidazole, VCM = vancomycin, WBC = white blood cell.

Despite liver abscess drainage, persistent fever, and no improvement in the WBC count or CRP level were observed (Fig. 3). The surgical treatment could not be performed because of the presence of DIC and poor general condition of the patient. The patient’s respiratory condition gradually worsened; consequently, he was admitted to the intensive care unit and put on ventilator support on day 6. Ascites and pleural effusion associated with renal failure and systemic inflammatory response syndrome also occurred, and continuous hemodiafiltration was initiated for renal failure on day 22. The patient’s general condition did not improve despite the ventilator support and continuous hemodiafiltration and he died on day 37.

**Figure 4.**
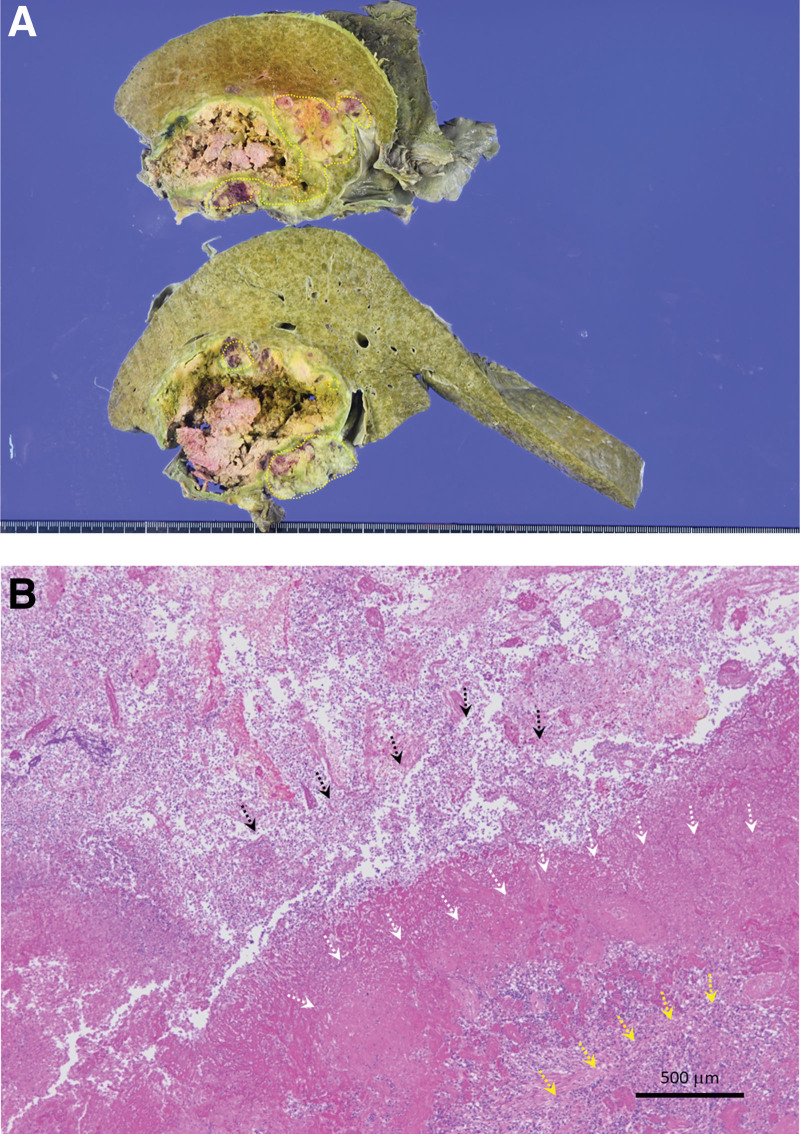
Findings of postmortem examination. (A) The cut surface showed tumor with marked necrosis. Residual tumor (yellow area) was observed in the peripheral lesion. (B) Histologically, tumor showed marked necrotic (white arrows), with residual HCC (yellow arrows) at the margins and an abscess (black arrows) at the center of the tumor.

Postmortem examination (Fig. 4) showed that approximately 80% of the tumor was necrotic, with residual HCC at the margins and an abscess at the center of the tumor. The abscesses spread to the surrounding liver, ascending colon, and serosa of the small intestine. No evidence of stones or cholangitis in the CBD was observed.

## 3. Discussion

Atezolizumab plus bevacizumab for unresectable HCC significantly prolonged overall and progression-free survival compared to sorafenib in a phase 3 trial^[5]^ and is used as the first-line treatment for unresectable HCC in Japan. Adverse events that were reported in over 10% of the patients in the phase 3 trial included hypertension, fatigue, proteinuria, hepatotoxicity, pruritus, diarrhea, constipation, nausea, abdominal pain, decreased appetite, weight loss, pyrexia, and rash^[5]^; however, no development of a liver abscess was observed. Additionally, the development of liver abscesses after atezolizumab plus bevacizumab treatment of unresectable HCC in clinical practice has not been reported.

The etiology of pyogenic liver abscesses can be divided into six categories: biliary infection (in the setting of malignancy, biliary stenting, choledocholithiasis, and malformations), portal vein seeding (diverticulitis and appendicitis), hepatic arterial seeding (indwelling catheter infection, endocarditis, and severe sepsis), direct extension (cholecystitis, perinephric abscess, and subphrenic abscess), penetrating trauma, and cryptogenic causes.^[6]^ Risk factors for pyogenic liver abscesses include malignancy, diabetes, ischemic heart disease, chronic obstructive pulmonary disease, cirrhosis, renal failure, and liver transplant.^[7]^ In the present case, CT showed CBD stones but no evidence of obstructive jaundice, such as IHBD to CBD dilation on CT or cholangitis in the CBD on postmortem examination.

The occurrence of liver abscesses after treatment with radiofrequency ablation or TACE for HCC has been reported in many cases, with a frequency of 1.5%–2.4% after radiofrequency ablation^[8]^ and 0%–3.3% after TACE.^[9]^ The development of liver abscesses after molecular-targeted therapies for HCC has been reported in recent years. Shin et al reported that liver abscesses developed within necrotic areas after sorafenib treatment for multiple HCCs with portal vein tumor thrombosis.^[10]^ There is a case report of liver abscess development after chemotherapy with bevacizumab, capecitabine, and oxaliplatin for colorectal carcinoma with liver and lung metastases.^[11]^ In this patient, bevacizumab, a vascular endothelial growth factor inhibitor, inhibited tumor angiogenesis, resulting in tumor necrosis and an anaerobic environment that facilitated the proliferation of anaerobic bacteria, leading to liver abscesses. In a retrospective study of patients with HCC treated with transarterial chemoembolization and molecular targeted agents with or without immune checkpoint inhibitors, tumor necrosis was more likely to occur in patients with larger tumor size and higher AFP levels.^[12]^ Risk factors for liver abscesses, such as malignancy and renal failure, and the large tumor size (11 cm) may have contributed to liver abscess development in this patient.

Gas-forming pyogenic liver abscess (GFPLA) accounts for 5.6%–31.8% of pyogenic liver abscesses. The mortality rate of GFPLA ranges from 25.7% to 37.1%, and its prognosis is poor.^[13]^ In the present case, *R. ornithinolytica* was detected in the puncture fluid of the liver abscess and is considered to be a pathogenic bacterium of the liver abscess. The most common GFPLA bacterial species is *Klebsiella pneumoniae*, followed by *Escherichia coli*, *Streptococcus* spp., and *Clostridium perfringens*^[14,15]^; however, only one case of *R. ornithinolytica* has been reported^[16]^ which is extremely rare. *R. ornithinolytica* is a capsulated Gram-negative rod that belongs to the Enterobacteriaceae family and is closely related to *Klebsiella* spp.^[17]^ Although a few cases of human infections caused by *R. ornithinolytica* have been reported, since 2015, these infections, including urinary tract infections, pneumonia, wound infections, catheter infections, meningitis, cholangitis, pancreatitis, and peritonitis, have been increasing.^[18]^ Risk factors for *R. ornithinolytica* infection include cancer, invasive procedures (urinary catheters, ventilator support, central venous catheters), immunodeficiency, diabetes, heavy alcohol consumption, and chronic kidney disease.^[18–20]^ Cancer and chronic kidney disease were the risk factors in the present case.

Additionally, GFPLAs are easy to perforate because they can more likely cause extensive liver tissue damage, fragile abscess walls, and increased intra-abscess pressure due to gas production.^[21]^ When the abscess perforates the abdominal cavity, surgical treatment is difficult because of the poor general condition of the patient. In the present case, percutaneous liver abscess drainage was performed immediately after hospital transfer. A few reports have described the development of liver abscesses after the initiation of the molecular targeted agents for unresectable HCC^[10,22]^; therefore, liver abscesses can develop after the initiation of molecular targeted agents that inhibit tumor angiogenesis. In addition, because common bile duct stones are a common cause of liver abscesses, their removal before commencing the HCC treatment could have prevented the development of liver abscesses.

In conclusion, we report the first case of a liver abscess after atezolizumab plus bevacizumab treatment for unresectable HCC. If necrotic areas appear within the tumor after initiation of atezolizumab plus bevacizumab treatment for unresectable HCC, liver abscesses may develop, especially in patients with the risk factors for liver abscess, as described above. The risk factors and mechanisms of liver abscess development after atezolizumab plus bevacizumab treatment in patients with unresectable HCC should be further investigated.

## Author contributions

Data collection: Naomi Uchiyama, Hiroshi Hatada, Kenichi Nakamura, Yuri Komaki.

Literature reviews and curation: Hisayoshi Iwakiri, Satoru Hasuike, Kenji Nagata, Yoshinori Ozono, Hiroshi Kawakami.

Postmortem examination: Yuichiro Sato.

Writing – original draft: Keisuke Uchida.

Writing – review & editing: Yoshinori Ozono, Hiroshi Kawakami.
